# Supplementary material for: The role of primary care providers in testing for sexually transmitted infections in the MassHealth Medicaid program
Source: PLoS One. 2023 Nov 30;18(11):e0295024. doi: 10.1371/journal.pone.0295024 (PMC10688870; doi:10.1371/journal.pone.0295024)
Supplement: S1 Table — Table includes procedure codes used to identify STI testing in the MassHealth population 13–64 years old in CY 2019. (PDF) [file pone.0295024.s001.pdf]

Table S1. Procedure codes defining the presence of testing for sexually transmitted infections.	
**Code**	**Description**
87491	Infectious agent detection by nucleic acid (DNA or RNA); Chlamydia trachomatis, amplified probe technique.
86631	Antibody; Chlamydia.
86632	Antibody; Chlamydia, IgM.
87110	Culture, chlamydia.
87270	Infectious agent antigen detection by direct fluorescent antibody technique; Chlamydia trachomatis.
87320	Infectious agent antigen detection by enzyme immunoassay technique, qualitative or semiquantitative, multiple step method; Chlamydia trachomatis.
87490	Infectious agent detection by nucleic acid (DNA or RNA); Chlamydia trachomatis, direct probe technique.
87492	Infectious agent detection by nucleic acid (DNA or RNA); Chlamydia trachomatis, quantification.
87810	Infectious agent detection by immunoassay with direct optical observation; Chlamydia trachomatis.
87590	Infectious agent detection by nucleic acid (DNA or RNA); Neisseria gonorrhoeae; direct probe technique
87591	Infectious agent detection by nucleic acid (DNA or RNA); Neisseria gonorrhoeae; amplified probe technique.
87592	Infectious agent detection by nucleic acid (DNA or RNA); Neisseria gonorrhoeae; quantification.
87801	Infectious agent, multiple organisms
87850	Infectious agent detection by immunoassay with direct optical observation; Neisseria gonorrhoeae.
86592	Syphilis test; qualitative (eg, VDRL, RPR, ART).
86593	Syphilis test; quantitative.

Table includes procedure codes used to identify STI testing in the MassHealth population 13-64 years old in CY 2019
