# Supplementary material for: The role of primary care providers in testing for sexually transmitted infections in the MassHealth Medicaid program
Source: PLoS One. 2023 Nov 30;18(11):e0295024. doi: 10.1371/journal.pone.0295024 (PMC10688870; doi:10.1371/journal.pone.0295024)
Supplement: S2 Table — Analyses include only those enrolled in MassHealth for at least 183 days in 2019. RxCG and DxCG scores were modeled with additional terms for splines at RRS values of 5 and 20 to model a non-linear relationship between RxCG and DxCG and the probability of having an STI-related encounter. Each unit increase is 1 standard deviation. (PDF) [file pone.0295024.s002.pdf]

Table S2a. Logistic regression output for model testing differences between type of health care plan, adjusting for SDOH model variables, and predicting the one-year prevalence of STI testing at any provider in MassHealth Managed Care Eligible Population ages 13-64 (N=740,471) in CY 2019

Logistic regression | Number of obs = 740,471
LR chi2(19) = 90787.37
Prob > chi2 = 0.0000
Log likelihood = -329714.78 | Pseudo R2 = 0.1210

sti_any	Odds ratio	Std. err.	z	P>z	[95% conf.	interval]
STI_PLAN						
PCCM	1.281452	0.018543	17.14	0	1.245619	1.318317
ACPP	1.318142	0.014477	25.15	0	1.29007	1.346825
Primary ACO	1.51881	0.017154	37	0	1.485559	1.552806
STI_agegrp						
13-18	0.4773109	0.004887	-72.24	0	0.4678282	0.4869858
25-44	0.4563414	0.004184	-85.57	0	0.448215	0.4646151
45-64	0.1245841	0.001469	-176.66	0	0.1217382	0.1274965
FEMALE	2.813573	0.019101	152.38	0	2.776385	2.85126
STI_HOUSINGgrp						
Unstable only	1.289966	0.012799	25.66	0	1.265123	1.315298
Homeless	1.580465	0.030372	23.82	0	1.522045	1.641127
STI_DISABILITY						
Other Disabled	0.7194771	0.007944	-29.82	0	0.7040745	0.7352168
DDS(notDMS)	0.4100354	0.010695	-34.18	0	0.3896002	0.4315425
DMS Client	0.7112882	0.023215	-10.44	0	0.667213	0.7582749
STI_NSS7*	1.181878	0.003607	54.75	0	1.174829	1.188969
STI_DxCG	1.286542	0.004507	71.92	0	1.277739	1.295406
STI_k5DxCG	0.6736894	0.003948	-67.4	0	0.6659956	0.6814721
STI_k20DxCG	1.277629	0.021973	14.25	0	1.23528	1.32143
STI_RxCG	1.178737	0.004125	46.99	0	1.17068	1.186849
STI_k5RxCG	0.8068327	0.004718	-36.7	0	0.7976377	0.8161337
STI_k20RxCG	1.08053	0.015957	5.24	0	1.049703	1.112263
_cons	0.1705452	0.002233	-135.08	0	0.166224	0.1749788

Notes: Analyses include only those enrolled in MassHealth for at least 183 days in 2019. RxCG and DxCG scores were modeled with additional terms for splines at RRS values of 5 and 20 to model a non-linear relationship between RxCG and DxCG and the probability of having an STI-related encounter.
*Each unit increase is 1 standard deviation.

Table S2b. Post-estimation contrasts testing differences between MassHealth health care plan types following the analysis represented in Table S2a.

	Odds ratio	Std. err.	z	P>z	[95% conf.	interval]
(MCO vs Primary Care ACO)	0.6584101	0.007436	-37	0	0.6439954	0.6731474
(PCCM vs Primary Care ACO)	0.8437212	0.009888	-14.5	0	0.8245619	0.8633258
(ACPP vs Primary Care ACO)	0.8678781	0.006014	-20.45	0	0.8561702	0.8797462

	Odds ratio	Std. err.	z	P>z	[95% conf.	interval]
(MCO vs ACPP)	0.7586435	0.008332	-25.15	0	0.7424869	0.7751516
(PCCM vs ACPP)	0.9721656	0.011075	-2.48	0.013	0.9506993	0.9941165
(Primary Care ACO vs ACPP)	1.152236	0.007985	20.45	0	1.136692	1.167992

	Odds ratio	Std. err.	z	P>z	[95% conf.	interval]
(MCO vs PCCM)	0.7803645	0.011292	-17.14	0	0.7585431	0.8028136
(ACPP vs PCCM)	1.028631	0.011718	2.48	0.013	1.005918	1.051857
(Primary Care ACO vs PCCM)	1.185226	0.01389	14.5	0	1.158311	1.212765
