# Supplementary material for: The role of primary care providers in testing for sexually transmitted infections in the MassHealth Medicaid program
Source: PLoS One. 2023 Nov 30;18(11):e0295024. doi: 10.1371/journal.pone.0295024 (PMC10688870; doi:10.1371/journal.pone.0295024)
Supplement: S3 Table — Analyses include only those enrolled in MassHealth for at least 183 days in 2019. RxCG and DxCG scores were modeled with additional terms for splines at RRS values of 5 and 20 to model a non-linear relationship between RxCG and DxCG and the probability of having an STI-related encounter. Each unit increase is 1 standard deviation. (PDF) [file pone.0295024.s003.pdf]

Table S3a. Logistic regression output for model testing differences between type of health care plan, adjusting for SDOH model variables, and predicting the one-year prevalence of STI testing at a primary care setting in MassHealth Managed Care Eligible Population ages 13-64 (N=740.417) in CY 2019

Logistic regression	Number of obs = 740,471
	LR chi2(19) = 43364.88
	Prob > chi2 = 0.0000
Log likelihood = -179760.18	Pseudo R2 = 0.1076

**_sti_any**	Odds ratio	Std. err.	z	P>z	[95% conf.	interval]
STI_PLAN						
PCCM	3.431804	0.0799456	52.93	0	3.278637	3.592127
ACPP	1.612927	0.0334374	23.06	0	1.548705	1.679813
Primary ACO	3.694204	0.0754263	64	0	3.54929	3.845035
STI_agegrp						
13-18	0.5403558	0.0076541	-43.45	0	0.5255604	0.5555677
25-44	0.4586042	0.0057339	-62.35	0	0.4475027	0.4699812
45-64	0.1324336	0.0023145	-115.68	0	0.1279741	0.1370486
FEMALE	2.60108	0.0271082	91.72	0	2.548488	2.654757
STI_HOUSINGgrp						
Unstable only	1.222127	0.0169468	14.47	0	1.189359	1.255798
Homeless	1.103753	0.0307921	3.54	0	1.045022	1.165785
STI_DISABILITY						
Other Disabled	0.8312234	0.013467	-11.41	0	0.8052432	0.8580418
DDS(notDMS)	0.4572328	0.0179572	-19.93	0	0.4233577	0.4938184
DMS Client	0.8523248	0.0384722	-3.54	0	0.78016	0.9311649
STI_NSS7*	1.126868	0.0050194	26.82	0	1.117073	1.136749
STI_DxCG	1.24983	0.0061896	45.03	0	1.237758	1.262021
STI_k5DxCG	0.7056206	0.0061091	-40.27	0	0.693748	0.7176963
STI_k20DxCG	1.314035	0.0315557	11.37	0	1.25362	1.377362
STI_RxCG	1.13784	0.005686	25.84	0	1.12675	1.149039
STI_k5RxCG	0.8388098	0.0071556	-20.6	0	0.8249016	0.8529524
STI_k20RxCG	1.055857	0.0237159	2.42	0.016	1.010383	1.103378
_cons	0.0318656	0.0007256	-151.35	0	0.0304748	0.03332

Notes: Analyses include only those enrolled in MassHealth for at least 183 days in 2019. RxCG and DxCG scores were modeled with additional terms for splines at RRS values of 5 and 20 to model a non-linear relationship between RxCG and DxCG and the probability of having an STI-related encounter. *Each unit increase is 1 standard deviation.

Table S3b. Post-estimation contrasts testing differences between MassHealth health care plan types following the analysis represented in Table S3a.

	Odds ratio	Std. err.	z	P>z	[95% conf.	interval]
(MCO vs Primary Care ACO)	0.2706943	0.0055269	-64	0	0.2600757	0.2817465
(PCCM vs Primary Care ACO)	0.9289699	0.013611	-5.03	0	0.9026722	0.9560338
(ACPP vs Primary Care ACO)	0.4366103	0.004382	-82.57	0	0.4281057	0.4452838

	Odds ratio	Std. err.	z	P>z	[95% conf.	interval]
(MCO vs ACPP)	0.6199907	0.012853	-23.06	0	0.5953043	0.6457008
(PCCM vs ACPP)	2.127687	0.0320338	50.15	0	2.065819	2.191407
(PrimaryCare ACO vs ACPP)	2.290372	0.022987	82.57	0	2.245759	2.335872

	Odds ratio	Std. err.	z	P>z	[95% conf.	interval]
(MCO vs PCCM)	0.2913919	0.0067881	-52.93	0	0.2783866	0.3050048
(ACPP vs PCCM)	0.469994	0.0070761	-50.15	0	0.4563277	0.4840695
(Primary Care ACO vs PCCM)	1.076461	0.015772	5.03	0	1.045988	1.107822
