# Supplementary material for: The role of primary care providers in testing for sexually transmitted infections in the MassHealth Medicaid program
Source: PLoS One. 2023 Nov 30;18(11):e0295024. doi: 10.1371/journal.pone.0295024 (PMC10688870; doi:10.1371/journal.pone.0295024)
Supplement: Attachment — Submitted filename: STI-CDC1_Response_Letter_20230908.docx [file pone.0295024.s004.docx]

TO:	Hamufare Dumisani Dumisani Mugauri, Ph.D. Public Health
Academic Editor
PLOS ONE

RE:	PONE-D-23-14033
The Role of Primary Care Providers in Testing for Sexually Transmitted Infections in the MassHealth Medicaid Program

September 8, 2023

Dear Dr. Mugauri,

In the following, please note that we have added character prefixes to your numbered points, to keep track of whether they were journal requirements (JR), or made by you, the editor (E), reviewer 1 (R1) or reviewer 2 (R2). E.g., the first point raised by the journal is now labeled JR1.

Journal Requirements:

When submitting your revision, we need you to address these additional requirements.

JR1. Please ensure that your manuscript meets PLOS ONE's style requirements, including those for file naming. The PLOS ONE style templates can be found at 
[https://journals.plos.org/plosone/s/file?id=wjVg/PLOSOne_formatting_sample_main_body.pdf](https://nam10.safelinks.protection.outlook.com/?url=https%3A%2F%2Fjournals.plos.org%2Fplosone%2Fs%2Ffile%3Fid%3DwjVg%2FPLOSOne_formatting_sample_main_body.pdf&data=05%7C01%7CEric.Mick%40umassmed.edu%7C880fa5821ae84bee835208db9360a63e%7Cee9155fe2da34378a6c44405faf57b2e%7C0%7C0%7C638265815554561671%7CUnknown%7CTWFpbGZsb3d8eyJWIjoiMC4wLjAwMDAiLCJQIjoiV2luMzIiLCJBTiI6Ik1haWwiLCJXVCI6Mn0%3D%7C3000%7C%7C%7C&sdata=cZQcDWqSibUVBmU0MRBZx%2FanEVkTwLgkrjvo54SCOAY%3D&reserved=0) and 
[https://journals.plos.org/plosone/s/file?id=ba62/PLOSOne_formatting_sample_title_authors_affiliations.pdf](https://nam10.safelinks.protection.outlook.com/?url=https%3A%2F%2Fjournals.plos.org%2Fplosone%2Fs%2Ffile%3Fid%3Dba62%2FPLOSOne_formatting_sample_title_authors_affiliations.pdf&data=05%7C01%7CEric.Mick%40umassmed.edu%7C880fa5821ae84bee835208db9360a63e%7Cee9155fe2da34378a6c44405faf57b2e%7C0%7C0%7C638265815554561671%7CUnknown%7CTWFpbGZsb3d8eyJWIjoiMC4wLjAwMDAiLCJQIjoiV2luMzIiLCJBTiI6Ik1haWwiLCJXVCI6Mn0%3D%7C3000%7C%7C%7C&sdata=O%2FvgeDj7kL3g6lOH7D%2Bd3ZCk2iWxOLcN0o3uoMoyYFA%3D&reserved=0)

We did this.

JR2. Thank you for stating the following financial disclosure: 
"This publication was supported by the Centers for Disease Control and Prevention of the U.S. Department of Health and Human Services (HHS) as part of a financial assistance award totaling $113,710 with 100 percent funded by CDC/HHS. The contents are those of the author(s) and do not necessarily represent the official views of, nor an endorsement by, CDC/HHS or the U.S. Government. "

Please state what role the funders took in the study.  If the funders had no role, please state: "The funders had no role in study design, data collection and analysis, decision to publish, or preparation of the manuscript." 
If this statement is not correct you must amend it as needed. 
Please include this amended Role of Funder statement in your cover letter; we will change the online submission form on your behalf.

The amended Funder statement is:

This study was originally funded by CDC, Division of STD Prevention, and the award was made to the CDC Foundation, which in turn subcontracted the work to UMass Chan. Dr. Pearson of the CDC participated in the conception, design, analysis, and overall management of the project. The contents are those of the author(s) and do not necessarily represent the official views of, nor an endorsement by, CDC/HHS or the U.S. Government.

JR3. Thank you for stating the following in the Acknowledgments Section of your manuscript: 
"This publication was supported by the Centers for Disease Control and Prevention of the U.S. Department of Health and Human Services (HHS) as part of a financial assistance award totaling $113,710 with 100 percent funded by CDC/HHS. The contents are those of the author(s) and do not necessarily represent the official views of, nor an endorsement by, CDC/HHS or the U.S. Government. "

We note that you have provided funding information that is not currently declared in your Funding Statement. However, funding information should not appear in the Acknowledgments section or other areas of your manuscript. We will only publish funding information present in the Funding Statement section of the online submission form. 
Please remove any funding-related text from the manuscript and let us know how you would like to update your Funding Statement. Currently, your Funding Statement reads as follows: 
"This publication was supported by the Centers for Disease Control and Prevention of the U.S. Department of Health and Human Services (HHS) as part of a financial assistance award totaling $113,710 with 100 percent funded by CDC/HHS. The contents are those of the author(s) and do not necessarily represent the official views of, nor an endorsement by, CDC/HHS or the U.S. Government. "

Please include your amended statements within your cover letter; we will change the online submission form on your behalf.

We apologize for the confusion. We removed the acknowledgement and changed the Funding Statement to the text just above, in JR2.

JR4. Please include captions for your Supporting Information files at the end of your manuscript, and update any in-text citations to match accordingly. Please see our Supporting Information guidelines for more information: [http://journals.plos.org/plosone/s/supporting-information](https://nam10.safelinks.protection.outlook.com/?url=http%3A%2F%2Fjournals.plos.org%2Fplosone%2Fs%2Fsupporting-information&data=05%7C01%7CEric.Mick%40umassmed.edu%7C880fa5821ae84bee835208db9360a63e%7Cee9155fe2da34378a6c44405faf57b2e%7C0%7C0%7C638265815554561671%7CUnknown%7CTWFpbGZsb3d8eyJWIjoiMC4wLjAwMDAiLCJQIjoiV2luMzIiLCJBTiI6Ik1haWwiLCJXVCI6Mn0%3D%7C3000%7C%7C%7C&sdata=hvBqvSi98G5dhZTq6oH3ysA%2BrntBomR2v54olqZ6QYM%3D&reserved=0).

We did this.

Additional Editor Comments
E1. Principal findings: Avoid using the term "significant" in a scientific paper, unless if it denotes statistical significance.

We revised the manuscript throughout; “significant” is now only used for statistical significance and is so designated.

E2. Conclusions: "explicit incentives for that testing incorporated into primary care settings" How sustainable is this beyond the funding duration for you to conclude effectiveness of the model based on its incentive-influenced results

Sustainability should not be an issue. MassHealth already requires its ACOs to: increase the total dollars paid to its primary care clinical teams; pay PC practices via capitation; and allocate capitation dollars differently based on PCC teams accepting greater responsibility (and being held accountable) for coordinating care to include an expanded set of services, such as on-site behavioral health counseling. MassHealth, or others with managed care programs, could do the same with STI care. By incentivizing the use of low-cost testing, the program might even save money.

E3. Methodology is not explicit: study design, sampling and sample size calculation, inclusion/exclusion criteria, study period, data collection tools, ethical clearances are all missing.

We apologize for insufficient detail in this regard, and added substantial new text to address it:

This study was determined to be not human subjects research by the University of Massachusetts Chan Medical School Institutional Review Board. [Page 6]

And:

Population

We studied managed-care-eligible members aged 13-64 years enrolled in a MassHealth managed care delivery system for at least 6 months during 2019. Children less than 13 years of age were excluded because there is little STI risk in this population. We also exclude data from the few members (contributing only about 3% of member-months) who were enrolled for less than 6 months, because they contribute too little data for determining (annual) testing prevalence. We examined 2019 data because it was the first full year of ACO program implementation in Massachusetts and was untainted by disruptions due to the COVID-19 pandemic. [Page 6]

And:

Analyses used Stata v17.0. We used logistic regression to separately predict each of two dichotomous outcomes: any STI testing and testing in a primary care setting. Model building was influenced by our ongoing work with MassHealth. Since 2016, MassHealth has used variables like these for risk adjusting total cost of care, for primary care sub-capitation, and in quality measurement. Here we used a modified step-down model building approach, beginning by using MassHealth’s most current modeling structure for calculating expected total cost, and then modifying, dropping, or adding predictors based on statistical significance or factors specific to the outcome of STI testing. [Page 8]

And:

To test for differences in testing between plan types, we augmented the logistic regression models, adding terms for plan type. Given that we studied nearly three-quarters of a million members, with 12% in MCOs and 32% in Primary Care ACOs, the power to detect a difference in proportion between these two groups (even if it is as small as 1% and using a Type 1 error rate of alpha = 0.001) exceeds 99%. [Page 11]

E4. There is no evidence of how authors dealt with potential confounders or effect modifiers (table 2)

We now say:

These multivariable logistic models are used solely to establish expected levels of STI testing; odds ratios for individual variables reflect associations that should not be interpreted as causal, and some are affected by collinearity with other included variables. [Page 9 and Table 2 legend]

**Comments to the Author**
JR5. Have the authors made all data underlying the findings in their manuscript fully available?
The [PLOS Data policy](https://nam10.safelinks.protection.outlook.com/?url=http%3A%2F%2Fwww.plosone.org%2Fstatic%2Fpolicies.action%23sharing&data=05%7C01%7CEric.Mick%40umassmed.edu%7C880fa5821ae84bee835208db9360a63e%7Cee9155fe2da34378a6c44405faf57b2e%7C0%7C0%7C638265815554561671%7CUnknown%7CTWFpbGZsb3d8eyJWIjoiMC4wLjAwMDAiLCJQIjoiV2luMzIiLCJBTiI6Ik1haWwiLCJXVCI6Mn0%3D%7C3000%7C%7C%7C&sdata=bLluOZT04oQG0FKdeBCmutsc9MAXIVcptegEiB7WOE4%3D&reserved=0) requires authors to make all data underlying the findings described in their manuscript fully available without restriction, with rare exception (please refer to the Data Availability Statement in the manuscript PDF file). The data should be provided as part of the manuscript or its supporting information, or deposited to a public repository. For example, in addition to summary statistics, the data points behind means, medians and variance measures should be available. If there are restrictions on publicly sharing data—e.g. participant privacy or use of data from a third party—those must be specified.

The data belong to MassHealth; we cannot make them publicly available. We now state this and provide information about how to apply to MassHealth for accessing the data.

Reviewer #1: Overall
R1.A The manuscript by Mick et al. describes prevalence of STI testing both generally and in primary care settings, within the MassHealth Medicaid programme in the United States. The background and methods were generally well written and concise. However, as someone not overly familiar with the United States system for healthcare financing, I did find some parts of the methods difficult to follow. Some brief definitions and assumption of no knowledge of such healthcare plans may help bridge the gap for international readers.

Thank you for the suggestion. We added this text in the introduction:

In the United States, Medicaid is a public insurance program for low-income families and individuals with disabilities … [Page 4]

Medicaid programs are administered locally by states but regulated by the federal government through the Centers for Medicare and Medicaid Services (CMS). Massachusetts has been allowed to innovate and evaluate novel healthcare delivery modalities since 1985.[8] The current work was conducted under the authority of the Independent Evaluation of the Massachusetts Medicaid and Children’s Health Insurance Program’s (i.e., MassHealth’s) 1115 Demonstration Waiver Extension (2017-2022). [Page 4]

R1.B The results were interesting, particularly how STI testing in primary care was apparently enhanced by the more primary-care focussed PCCM and primary care PCO plans. However, I feel that more could be said in the discussion about the limitations of using such large datasets, without specific indicators for STI risk, particularly when assessing associations between healthcare plan and STI testing.

We agree that understanding risk/need for STI testing is important, and that our data say little about it. Indeed, learning the limitations of the existing data for documenting need and using evidence of testing to understand STI surveillance was part of our purpose. We now say:

Administrative data (claims and encounter records) tell us little about an individual’s level of need for STI testing (e.g., due to the presence or absence of risky behaviors, or STI-like symptoms). Also, although we would like to learn about how organizational structures affect the comprehensiveness of STI surveillance, we cannot distinguish between tests done for diagnosis or for screening (in the absence of symptoms), nor can we find non-testing activities that have ruled out the need for testing. Thus, this study examines STI testing, an imperfect but useful proxy for STI surveillance activity. [Page 4-5]

… This study sought to: 1) determine the annual prevalence of STI testing both overall and specifically within primary care settings, 2) identify demographic, clinical, and social characteristics that predict STI testing, and 3) explore whether testing is more prevalent for members of ACOs than for those enrolled in other types of health plans. [Page 5]

Also:

Our findings should be considered in the context of study limitations. Most important is the reliance on claims and encounter data to estimate the prevalence of STI testing, and more particularly, testing by a primary care clinical practice. While our data show where STI testing was conducted – or, more specifically, the place cited in bills – it does not reveal who ordered the test or its result. We relied on administrative files provided by the state to determine if a particular encounter was in a primary care setting. Our finding that the plans billing MassHealth directly (i.e., the PCCM plan and Primary Care ACOs) had more primary-care testing than the MCO and ACPP ACO plans (that receive capitation payments from MassHealth and submit encounter records) may at least partially reflect how primary care activity is reported and not necessarily how it is delivered. An additional study limitation is that we do not see sexually transmitted disease care sought outside of MassHealth for reasons of confidentiality. [Page 13]

Background
R1.1 “As of 2019, over two-thirds of MassHealth’s one million eligible members were enrolled in accountable care organizations (ACOs) as part of an 1115 Demonstration Waiver. Through this program, MassHealth requires ACOs to engage frontline primary care practices, using value-based payments tied to cost and quality performance as incentives.”
The authors assume reader knowledge of United States healthcare financing. To enable a more international audience to benefit from reading the paper, I suggest not assuming such knowledge. This includes, for example, including a brief definition of an “accountable care organization”, explaining what an “1115 demonstration waiver” entails, and generally providing basic descriptions of similar terms.

We added definitions for U.S. specific terms and categories:

Briefly, an MCO is a type of managed healthcare plan that is intended to reduce healthcare costs by providing various economic incentives to patients and providers to select less costly forms of care and by reviewing services for medical necessity. In contrast, ACOs are groups of providers who accept responsibility for the health and costliness of a population of attributed enrollees, with ACO and provider payment linked to performance on quality metrics and meeting total cost of care benchmarks. Members enrolled in MassHealth MCOs have access to just those providers in their MCO’s network. Members enrolled in the MassHealth PCCM plan have access to MassHealth’s entire network of providers, with their PCC responsible for coordinating their care. There are two predominant types of ACOs: Accountable Care Partnership Plan (ACPP) ACOs and Primary Care ACOs. ACPP ACOs are integrated partnerships of a provider-led ACO and an MCO which together serve as both the health plan and the provider system for their members. Primary Care ACOs are provider-led organizations contracting directly with MassHealth (that is, without an MCO) to deliver coordinated care and manage population health. [Page 7]

Methods
R1.2. “The outcome was presence of at least one test for an STI during 2019.”
Please provide a definition of “STI” here. I note that the procedure codes for defining the presence of STI testing are in the supplementary material but think it is helpful to have in the main manuscript.

We added the codes to the manuscript, leaving their descriptions in the supplemental material. We now say:

We identified claims or encounters for STI testing for each member using lists of procedure, revenue, and place of service codes that identify a testing visit (using HPC codes 87491, 86631-2, 8711, 87270, 87320, 87490, 87492, 87810, 87590, 87591-2, 87801, 87850, and 86592-3; see Supplemental Table S1 for code descriptions). [Page 6]

R1.3. “MassHealth managed care eligible members may choose to enroll in, or accept assignment to, one of three forms of managed care: 1) a managed care organization (MCO), 2) MassHealth’s PCC Case Management Plan (PCCM), or 3) an ACO.”
Similarly to above, please provide definitions for MCO and PCCM.

See response to R1.B.

R1.4. Analysis strategy
There does not appear to be an explicit analysis strategy. It seems that all the variables available were put into the model. Please provide justification for this.

Thank you for the suggestion – we added methodological detail, as quoted in E3, above.

Results
R1.5. “MassHealth members screened for STIs were more likely to be female, between the ages of 13 and 44, have unstable housing, live in more stressed neighborhoods, and have greater medical acuity, whether measured from diagnoses or pharmacy prescriptions.”
The age ranges are very broad, leading to a very heterogenous population within each category and making interpretation difficult. For example, 13-44 years includes both adolescents and young adults (who will likely have higher STI risk) as well as older individuals up to age 44. I note that the discussion also includes information on STI risk in different age groups (15-24, 25-34 etc) – it would be helpful to be able to compare more directly using the data in this paper.
I suggest using more narrow age categories. If these age groups are the only data available, then please state this as a limitation.

Thank you for this suggestion. We now use more meaningful age categories (see Table 1) and redesigned the tables to enable these comparisons.

R1.6. “MassHealth members screened for STIs were more likely to be female, between the ages of 13 and 44, have unstable housing, live in more stressed neighborhoods, and have greater medical acuity, whether measured from diagnoses or pharmacy prescriptions.”
Suggest including the unadjusted odds ratios and confidence intervals here.

We agree that unadjusted information about differences in rates would be of interest. Thus, we added observed testing rates for each categorical variable in Table 2, from which odds ratios can be calculated, if desired. These full-population descriptive statistics do not require CIs.

R1.7. Table 1The totals for housing problems and disability status add up correctly in the total, no STI testing, and STI testing columns. However, those for age do not, as follows:
Total: 352,743 + 527,851 + 212,549 = 1,093,143 [stated total = 1,093,149]
No STI testing: 352,293 + 477,244 +205,941 = 1,035,478 [stated total = 940,508]
STI testing: 450 +50,607 + 6,608 = 57,665 [stated total = 152,641]
Please rectify so that numbers are correct. If missing data, please state this.

This comment is no longer relevant given the new analyses. We checked the new tables for internal consistency.

R1.8. Table 1
I feel that row percentages would be helpful for the “no STI testing” and “STI testing” columns, rather than column percentages. For example, given that presence of at least one test for an STI is the outcome, I would argue that knowing the percentage of females (and males) who had at least one test provides more fruitful information than knowing that 50% of those who didn’t test and 74% of those who did test were female.
I acknowledge that mixing row and column percentages in a single table is not a good idea, so wonder if it might be possible to have a table purely dedicated to member characteristics, and another table showing the percentage of each variable (e.g. age group, sex) who did have an STI test.

Thank you for these comments. Table 1 now shows distributions of member characteristics and testing prevalence, overall and by plan type, with clear labeling as to what each percentage means.

R1.9. Table 1
Very minor point, but I found it a bit odd to state “%” and then use decimals. Would suggest if stating percentages, to state e.g. 32% rather than 0.32. My instinct was to read it as 0.32%.

Thank you – you are right; we corrected this throughout.

R1.10. Table 2
I would have thought there would be a risk of collinearity including both DxCG and RxCG in the same model. Was there any evidence of this?

The scores are, indeed, highly correlated. However, MassHealth typically includes both in its models because they provide qualitatively different, and complementary, information without creating problems in interpretability. In addition, given that our purpose in modeling here is for prediction, collinearity is not a concern (See our new text in E4, above).

Discussion
R1.11. “We found that STI testing was more prevalent among members with housing problems, greater medical morbidity, and living in more stressed neighborhoods, but less prevalent among members with disability.”
The main table that I feel is missing from the results and would be incredibly valuable would be a breakdown of characteristics (age, sex, disability status etc) between the different schemes (i.e. MCO, PCCM, ACPP, primary ACO). This would go some way to addressing one of my comments below about the differences between individuals enrolled in different schemes, and how this may affect STI testing. I appreciate these parameters are included in the models that helped you work out the observed/expected ratios, but would be helpful for the reader to see. This breakdown could be included in a new table 1 (if following my suggestion above).

As mentioned above, we appreciate the suggestion and agree. These edits have been incorporated into Table 1.

R1.12. “Primary among these is the reliance on claims and encounter data to estimate the prevalence of STI testing, and even more particularly, testing by a primary care clinical team. While our data show where STI testing was conducted – or, more specifically, the place cited in bills – it does not reveal who ordered the test or its result. We also relied on administrative files provided by the state to determine if a particular encounter was in a primary care setting. Our finding that the plans billing MassHealth directly (i.e., the PCCM plan and primary care ACOs) had more primary-care testing than the MCO and ACPP ACO plans (that receive capitation payments from MassHealth and submit encounter records) at least partially reflects technical artifacts related to how primary care activity is reported and not necessarily how it is delivered.
It is noted that plans billing MassHealth directly had more primary-care testing than the MCO and ACPP ACO plans, and how this is related to how primary care activity is reported. However, could such differences in reporting have biased the results? Could underreporting be a factor in some schemes but not others? Furthermore, are there any indicators as to the gross reliability of the underlying data?

We now address these issues in discussing study limitations(see R1.B and E4, above).

R1.13. There should be more discussion on the potential for confounding in influencing the differences in STI testing between different schemes, which I feel is the key limitation of this study. There is a relatively small number of variables that are adjusted for, and key variables such as presence of STI symptoms, recent contacts, or other indicators of sexual risk are not available. The variables that are included are quite general. As a result, it is difficult to really know whether it is the programmes themselves that are influencing STI testing, or whether those enrolled in different schemes have different STI risk profiles (which thus drives differences in STI testing).I do think that it is likely that the schemes are influencing STI testing (particularly regarding STI testing in primary care for the more primary care-focussed plans). However, at present the discussion doesn’t fully address the limitations of using such data.

These are all good points, that we address with new text at R1.B and E4, above. Also, we now describe the predictive power of the models:

We measure each model’s ability to predict testing using the C-Statistic (AUC or Area Under the Receiver Operating Characteristic curve) and by comparing observed rates of testing in the highest- versus lowest- deciles of model-predicted risk. [Methods, page 8]

and, in the results, point out that:

Our models had acceptable explanatory power (C-statistics of 74% and 72%) and could identify populations with very different levels of testing. Observed testing was 52.1% vs 4.8% in the top- vs. bottom-decile of model-predicted risk for testing by any provider, and 21.2% vs 1.5% for testing by a primary care provider. [Page 9]

Reviewer #2:

R2.1 Why was the clinic data of infants and children used in this analysis? That is not clear. It seems the primary outcome measure was of STI testing done in "adult primary care" sites in MA for people with Medicaid. Knowing the prevalence of testing of adolescents, young adults, and older adults (13-75 for example) would be more beneficial than using the number of persons ages 0-64.

We agree and now exclude members less than age 13. As described above, we made additional improvements in how we categorize age and sex for both modeling and reporting.

R2.2 Both concepts, STI screening and STI testing, are used without operational definitions. Are screening and testing equivalent concepts or is screening the sexual history taking and testing the labs ordered?

We revised the manuscript throughout to clarify that what we have studied is STI testing, which is an imperfect proxy for disease surveillance. See text above in answer to query R1.B.

R2.3 There is one mention of testing for GC, CT, and syphilis in the introduction, but not in the methodology and results. Was it just GC/CT/syphilis testing or was HCV, trich, and other STI testing as well?

We studied just GC, CT, and syphilis testing, to align with prior publications. We revised the manuscript to be clear about this, now including the codes used in the main body of the paper.

R2.4 Also, in the discussion, I'd suggest adding something to address the higher rate of GC (and probably syphilis as well) among men in MA, but that females were more likely to be tested. Adolescents and young adults have the highest age-group incidence rates of STIs, and this is mentioned, but nothing about men who have sex with men (MSM).

We have little insight into the underlying need for STI testing as we describe in our discussion of limitations and methods. Also, because these are administrative data we have no information on gender identity or sexual orientation. We now place our findings in context as follows:

Previous studies noted that Medicaid members were more likely to be identified as engaging in high-risk sexual behaviors if they were female, and between ages 15-24 and 25-34 (as compared to those aged 35-44 and 45-60).[24] MassHealth members more likely to be tested for STIs in this study were between 19 and 24 years of age, female, had housing problems or lived in higher stress neighborhoods, and had greater medical morbidity. [Page 12]

We now say:

Our finding that about 20% of MassHealth members aged 13-64 received STI testing is somewhat higher than findings in Medicaid populations (aged 0-64) in Maryland, where testing occurred in 16-17% and, quite a bit higher than in South Carolina, with testing in 10-11% of Medicaid members. [6,23] Since the Maryland and South Carolina studies did not exclude members under the age of 13, the comparable prevalence calculation in Massachusetts is 14 %.

R2.5 I'd also bring up in the introduction and discussion something about the diagnosis and treatment of an STI at the same time testing was done. Most GC, CT, and syphilis infections are asymptomatic. Hence, routine STI testing is recommended for those with higher-risk histories (new sexual partner, 2 or more partners, substance use before sex).
Otherwise it's not clear what percent of those tested were for routine screening (asymptomatic) or not.

We added discussion of the lack of insight into the need for testing or the underlying factors associated with screening overall. This is now included in both the introduction and the discussion.
