# Supplementary material for: The role of primary care providers in testing for sexually transmitted infections in the MassHealth Medicaid program
Source: PLoS One. 2023 Nov 30;18(11):e0295024. doi: 10.1371/journal.pone.0295024 (PMC10688870; doi:10.1371/journal.pone.0295024)
Supplement: Attachment — Submitted filename: Second_response_letter_20231107.docx [file pone.0295024.s005.docx]

TO:	Hamufare Dumisani Mugauri, PhD
Academic Editor, PLOS ONE

RE:	PONE-D-23-14033R1

The Role of Primary Care Providers in Testing for Sexually Transmitted Infections in the MassHealth Medicaid Program

November 7, 2023

Dear Dr. Mugauri,

Thank you for considering our manuscript for publication with PLOS ONE. We have addressed the additional editor and reviewer comments as follows:

**Additional Editor Comments:**

Please provide all the data used in this study as required by the journal

We apologize for the confusing response to this request in the first revision. We cannot share these data ourselves as access is controlled by the state of Massachusetts, who owns the data. It is possible to request these data from the Center for Health Information Analysis and we have provided their contact information.

We now say:

“These data belong to the Massachusetts Medicaid and Children's Health Insurance Program (MassHealth) and the research team cannot make these data publicly available themselves due to legal restrictions related to the use of data from a third party. If interested in requesting access to these data independently, researchers may contact the Massachusetts Center for Health Information Analysis (CHIA) through the data request portal (https://www.chiamass.gov/non-government-agency-apcd-requests).”

**Comments to the Author**

6. Review Comments to the Author

Reviewer #1: Thank you very much to the authors for their detailed and thoughtful responses. I only have a few additional minor suggestions for improved clarity:

1. Page 5: code 8711 should be 87110

Thank you this has been corrected.

2. “Our primary outcome was presence of at least one test for an STI during 2019. We
identified claims or encounters for STI testing for each member using lists of procedure, revenue, and place of service codes that identify a testing visit (using HPC codes 87491, 86631-2, 8711, 87270, 87320, 87490, 87492, 87810, 87590, 87591-2, 87801, 87850, and 86592-3; see Supplemental Table S1 for code descriptions).”

The definition of the primary outcome should be easily obtainable from the main paper, without referral to supplemental material. For example, “Our primary outcome was presence of at least one test for an STI (chlamydia, gonorrhea and/or syphilis) during 2019.”

Thank you for the suggestion which we have adopted:

Our primary outcome was presence of at least one test for an STI (chlamydia, gonorrhea and/or syphilis) during 2019. We identified claims or encounters for STI testing for each member using lists of procedure, revenue, and place of service codes that identify a testing visit (using HPC codes 87491, 86631-2, 87110, 87270, 87320, 87490, 87492, 87810, 87590, 87591-2, 87801, 87850, and 86592-3; see Supplemental Table S1 for code descriptions).

3. "Here we used a modified step-down model building approach, beginning by using MassHealth’s most current modeling structure for calculating expected total cost, and then modifying, dropping, or adding predictors based on statistical significance or factors specific to the outcome of STI testing.

a) What was the cut-off for “statistical significance” to modify, drop, or add predictors?
b) What were the starting variables in the model? (i.e. "MassHealth's most current modelling structure"

We’ve added the following to the description of the modeling:

“Here we used a modified step-down model building approach, beginning by using MassHealth’s most current modeling structure (i.e. age and sex, homelessness, unstable housing, disability status, the NSS7, and the DxCG and RxCG variables described above) for calculating expected total cost, and then modifying, dropping, or adding predictors based on statistical significance (p>0.05) or factors specific to the outcome of STI testing.”

4. Table 2

The “N” column is slightly confusing, as the first “ALL” row refers to all participants, but the subsequent rows refer to the number of participants in that category who tested. Perhaps an n/N column would be more appropriate? (i.e. number of people in that category who tested for STIs/total number of people in that category) e.g. 38461/333091 for “male” testing by any provider.

Thanks for the suggestion and we agree that it was a little confusing. Since the first ALL row only provided redundant information we’ve removed it. It was very busy with the n/N for each cell it would be required. So we added a column with totals for each subgroup to make the denominator clear.

Thank you again for the very thoughtful reviews.

Best regards,

Eric Mick, ScD

Department of Population and Quantitative Health Sciences

UMass Chan Medical School

55 Lake Avenue North

Worcester, MA 01655

USA
